# Elevated serum ferritin levels are associated with severity and prognosis of severe acute pancreatitis: a preliminary cohort study

**DOI:** 10.1186/s12876-022-02446-z

**Published:** 2022-09-05

**Authors:** Jie Wang, Qing-xie Liu, Dong-ling Teng, Yan-bing Ding, Guo-tao Lu, Wei-juan Gong, Qing-tian Zhu, Fei Han, Wei-ming Xiao

**Affiliations:** 1 Department of Gastroenterology, Pancreatic Center, The Affiliated Hospital of Yangzhou University, Yangzhou University, Yangzhou, 225000 Jiangsu China; 2 Yangzhou Key Laboratory of Pancreatic Disease, Institute of Digestive Diseases, The Affiliated Hospital of Yangzhou University, Yangzhou University, Yangzhou, 225000 Jiangsu China

**Keywords:** Acute pancreatitis, Serum ferritin, Organ failure, Retrospective study

## Abstract

**Background:**

Serum ferritin (SF), as an acute-phase response protein, is used to reflect the degree of oxidative stress and systemic inflammatory responses. This study was designed to assess the effect of elevated SF levels on the severity of acute pancreatitis (AP).

**Methods:**

From January 2013 to December 2020, 200 consecutive patients with AP were retrospectively reviewed to analyze the relationships among the etiologies of pancreatitis, the severity of the disease and SF levels. The receiver operating characteristic (ROC) curve and logistic regression analysis were used to assess whether elevated SF levels could predict the onset of organ failure in AP.

**Results:**

92 (46%) had high SF levels (> 275 ng/ml). SF levels were not associated with the etiology of AP disease. Among patients with high SF levels, there was a significant increase in the proportion of patients with severe AP (23.1% vs. 76.9%) and a higher proportion of systemic inflammatory response scores (25.9% vs. 44.6%) in comparison to patients with normal SF levels. The area under the ROC curve for SF in predicting persistent organ failure was 0.812 [95% confidence interval 0.721–0.904].

**Conclusions:**

F concentrations were positively correlated with the severity of AP, and quantitative assessment of SF can predict disease severity and organ failure in patients with AP.

**Supplementary Information:**

The online version contains supplementary material available at 10.1186/s12876-022-02446-z.

## Introduction

Acute pancreatitis (AP) is an acute inflammatory disease of the pancreas that can be caused by various conditions, such as cholelithiasis, hypertriglyceridemia, alcoholism, cancer and surgery [1]. AP patients can be divided into mild acute pancreatitis (MAP), moderate severe acute pancreatitis (MSAP) and severe acute pancreatitis (SAP) classifications according to local or systemic complications and the existence of transient or persistent organ failure [2–5]. SAP is characterized by organ failure that develops and persists > 48 h (known as persistent organ failure). In all AP patients, 10% to 20% of cases may develop persistent organ failure, with a patient mortality rate ranging from 10 to 30% [6, 7]. Early identification of patients with severe pancreatitis and persistent organ failure is important to guide clinical treatment decisions.

Serum ferritin (SF) is a soluble tissue protein that stores iron in the body and more than 90% of iron is bound to protein [8]. SF has many important biological functions [9], for example, SF can be used as an indicator of the intracellular storage of iron; it provides iron for hemoglobin synthesis in the bone marrow and is released as iron into the serum according to the needs of the body. Moreover, SF can also be used as an acute-phase protein and is widely used in the diagnosis and monitoring of diseases, such as adult-onset Still's disease, systemic lupus erythematosus, Hodgkin's lymphoma, arteriosclerosis, insulin resistance, acute infection and other diseases [10–12]. Interestingly, it has been found that serum ferritin is correlated with the severity of many diseases. There is no doubt that high serum ferritin levels are associated with many inflammatory diseases. It is inferred that iron induced hydroxyl radical formation leading to oxidative damage may be the contributing factor of inflammatory diseases [13–15]. Suárez-Santamaría et al. [16] found that SF levels was one of the most closely related variables of 28 day mortality in sepsis patients. However, the relationship between SF and AP remains unclear and has not been investigated thoroughly up to now.

The purpose of this study was to determine the association of serum SF levels with the severity of AP and to investigate whether elevated SF could predict the occurrence of organ failure in patients in the early stages of AP.

## Materials and methods

### Patient population and data collection

This study included 200 AP patients who were treated in the Affiliated Hospital of Yangzhou University from January 2013 to December 2020. According to the 2012 Atlanta classification of acute pancreatitis [5], patients with AP were diagnosed according to the following criteria: acute abdominal pain, elevated serum amylase (more than three times the upper limit of the normal range) and an imaging study with characteristic changes (CT, MRI, abdominal ultrasound or endoscopic ultrasound). The achievement of two criteria is required for a diagnosis of pancreatitis. The exclusion criteria for the study were as follows: for patients who were hospitalized two or several times within three months, only the initial results were included; patients younger than 18 years of age or older than 80 years of age; pregnant women; serious underlying diseases affecting the detection of SF, such as a history of renal failure; and patients with pancreatic tumors.

The basic information and clinical data (AP diagnosis, etiology, underlying diseases, serological indicators, local complications, organ failure, etc.) of AP patients were recorded. SIRS, Ranson’s, BISAP and CTSI scores within 48 h after admission were evaluated.

### The severity of acute pancreatitis, complications and the definition of persistent organ failure

AP is divided into MAP, MSAP and SAP according to the Atlanta classification [5]. MAP is not associated with organ failure or local/systemic complications, MSAP is associated with transient organ failure and/or local/systemic complications within 48 h and SAP can be accompanied by persistent organ failure over more than 48 h and can involve one or more organs. Persistent organ failure was defined by a modified Sequential Organ Failure Assessment (SOFA) score of at least 2 for 48 h or more that manifested in failure of at least one organ system; i.e., respiratory, cardiovascular or renal systems [17, 18]. Local complications include acute peripancreatic fluid collection (APFC), pancreatic pseudocyst, acute necrotizing collection (ANC) and walled-off necrosis (WON). Systemic complications include respiratory failure (such as ARDS), circulatory failure, renal failure [5, 19].

### Serum ferritin analysis

Venous blood samples (3–5 ml) were collected within 72 h from attack and 24 h after admission, and the serum was cleared within 2 h. The ARCHITECT Ferritin Reagent Kit was used to quantitate human SF and was supplied by Abbott Laboratories. According to the manufacturer's instructions, SF level > 275 ng/ml was defined as a positive test.

### Statistical analysis

Based on the information available for the general population, the underlying disease, etiology and severity of AP, personal history and clinical results were compared with respect to SF levels. First, patients with high SF levels were compared to patients with normal SF levels. Statistical analysis was performed using SPSS software (version 16.0 SPSS) and GraphPad Prism. Continuous variables were reported as the means and standard deviations based on the distribution of the variables. Categorical variables were described using percentages. The statistics were analyzed using the nonparametric rank-sum test, the chi-square test, Fisher’s exact test and Student’s t-test. Related analyses were performed using Pearson’s simple correlation. The receiver operating characteristic (ROC) curve was constructed for the predictor variables. The area under the curve (AUC) and 95 percent confidence intervals (CI) were calculated. And comparing ROC curves, AUC values, and *P* values of ferritin with other possible predictors (BISAP scores; WBC; AST; GGT; Cr). Optimal cut-off values for sensitivity and specificity for each parameter were derived from the ROC curves. Logistic regression analysis was conducted to evaluate the risk factors for poor clinical outcomes. All tests were two-tailed, and statistical significance was set at *P* < 0.05.

## Results

### Patient demographics and clinical characteristics

In total, 200 consecutive patients with AP who fulfilled the inclusion criteria were enrolled in this study. As shown in Table 1, we compared the severity of AP at different SF levels. The results showed that the proportion of SAP patients with elevated SF was higher (10.9% vs. 2.8%, *P* < 0.001), in addition, the proportion of patients who met the SIRS criteria was higher compared to patients with normal SF levels (44.6% vs. 25.9%, respectively, *P* = 0.006). Furthermore, the Ranson’s (2.0 vs. 1.0, *P* = 0.011), CTSI (3.1 vs. 2.3, *P* < 0.001) and BISAP scores (1.3 vs. 0.5, *P* < 0.001) were also higher in AP patients with elevated SF than in patients with normal SF levels.

**Table 1:** Comparison of Clinical characteristics and outcomes between AP patients with versus without high serum Ferritin levels

Variable	Overall	Normal SF	High SF	*P* value
	N = 200	N = 108	N = 92	
Age (mean ± sd), yrs	57.1 ± 15.7	56.3 ± 15.3	57.8 ± 16.3	0.503
Male sex, N (%)	110 (55%)	44 (40.7%)	66 (71.7%)	< 0.001
Smoking, N (%)	43 (21.5%)	20 (18.5%)	23 (25%)	0.303
Drinking, N (%)	29 (14.5%)	13 (12.0%)	16 (17.4%)	0.301
Weight (mean ± sd)	70.6 ± 12.3	67.6 ± 12.0	73.4 ± 12.1	0.021
*Underlying disease, N (%)*				
Diabetes	19 (9.5%)	8 (7.4%)	11 (12.0%)	0.285
Hypertension	30 (15%)	14 (13.0%)	16 (17.4%)	0.385
Coronary heart disease	13 (6.5%)	6 (5.6%)	7 (7.6%)	0.560
Fatty liver	26 (13%)	15 (13.9%)	11 (12.0%)	0.687
Etiology, N (%)				0.094
Biliary	101 (50.5%)	51 (47.2%)	50 (54.3%)	
Alcohol	14 (7%)	8 (7.4%)	6 (6.5%)	
Hypertriglyceridemia	41 (20.5%)	19 (17.6%)	22 (23.9%)	
Others	44 (22%)	30 (27.8%)	14 (15.2%)	
Severity, N (%)				< 0.001
MAP	149 (74.5%)	94 (63.1%)	55 (36.9%)	
MSAP	38 (19%)	11 (28.9%)	27 (71.1%)	
SAP	13 (6.5%)	3 (23.1%)	10 (76.9%)	
SIRS, N (%)	69 (34.5%)	28 (25.9%)	41 (44.6%)	0.006
Ranson score	1.5 ± 1.0	1.0 ± 0.9	2.0 ± 1.0	< 0.001
CTSI score	2.1 ± 1.2	1.7 ± 0.9	2.6 ± 1.4	< 0.001
BISAP score	0.9 ± 0.7	0.5 ± 0.6	1.3 ± 0.7	< 0.001
Local complications, N (%)	28 (14%)	8 (7.4%)	20 (21.7%)	0.005
Systemic complications, N (%)	10 (5%)	3 (2.8%)	7 (7.6%)	0.133

Data are presented as the means ± standard deviation or N%. *P* values were determined by Student’s t-test for continuous variables and the chi-square test for categorical variables. *P* < 0.05 was considered statistically significant

### The relationship between SF levels and AP disease severity

**Fig. 1:**
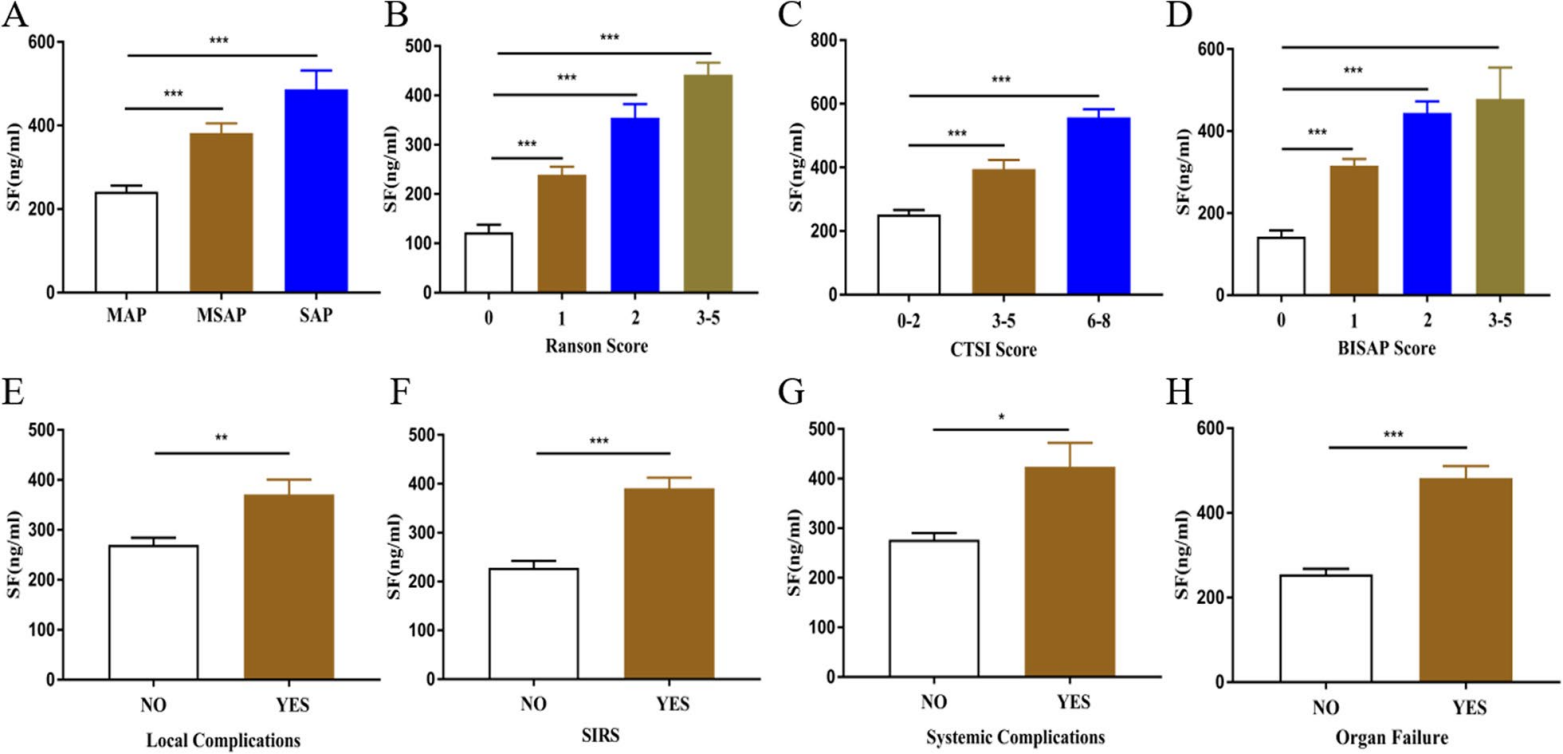
Comparison of mean serum Ferritin concentrations by Ranson score, CTSI score, BISAP score, SIRS, Local complications, systemic complications and Persistent organ failure

Firstly, the levels of SF according to the severity of disease were observed. As shown in Fig. 1A, it is self-evident that there was a significant progressive increase of SF in MAP, MSAP and SAP patients according to the Atlanta type (*P* < 0.001). What's more, the analysis of the relationship between SF level and different clinical disease severity scores (Ranson score, CTSI score and bisap score) found that the higher the score, the higher the SF level, shown in Fig. 1B–D.

Secondly, the levels of SF between patients with local or/and systemic complications (SIRS and persistent organ failure) and those without complications were compared. As shown in Fig. 1E–H, compared with patients without complications, SF levels in patients with complications (local or persistent organ failure or SIRS) were significantly increased.

**Fig. 2:**
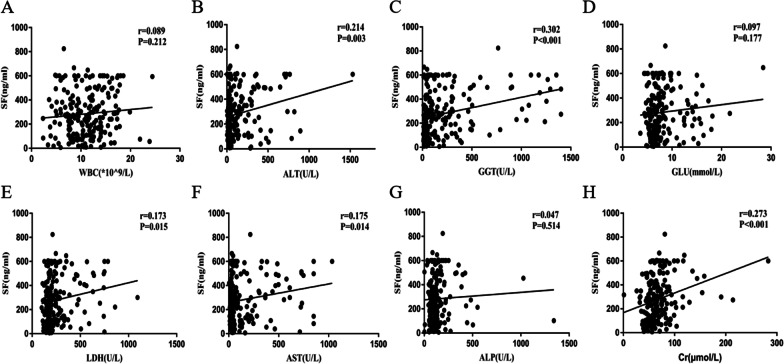
Correlation between SF and other clinical indicators in AP patients

Finally, the correlation between SF and other serological indicators were analyzed. As shown in Fig. 2, SF levels correlated positively with alanine aminotransferase (ALT, r = 0.214, *P* = 0.003), aspartate transaminase (AST, r = 0.175, *P* = 0.014), glutamyl transpeptidase (GGT, r = 0.302, *P* < 0.001), serum lactate dehydrogenase (LDH, r = 0.173, *P* = 0.015) and creatinine (Cr, r = 0.273, *P *< 0.001), and no significant correlations with white blood cell (WBC), alkaline phosphatase (ALP) or serum glucose (GLU) values were observed.

All of the above results showed a positive correlation between SF levels and the severity of AP disease.

### Logistic regression analysis

Logistic regression analysis was performed to evaluate the risk factors for disease severity and organ failure of AP patients (Table 2). No significant associations were found between SAP patients and Age, Drinking, Smoking, Diabetes, Fatty Liver, Coronary Heart Disease, WBC. Meanwhile, patients with organ failure and Male, Smoking, Diabetes, Fatty Liver, Coronary Heart Disease, WBCs were also no significant associations. Thus, logistic regression analysis indicated that serum SF was independent risk factors for severe acute pancreatitis and organ failure in patients with AP with odds ratios (ORS) of 1.009 (95% CI 1.004–1.014) and 1.011 (95% CI 1.006–1.015) respectively. Interestingly, we further studied the association between specific organ failure and serum SF, and found that serum SF was also the independent risk factor for acute kidney injury (AKI) and acute respiratory distress syndrome (ARDS) (Additional file 1: Table S1).

**Table 2:** Logistic regression analysis of severe acute pancreatitis and organ failure in patients with AP

Variable	B	OR	95% CI	*P* value
*SAP patients (N* = *13)*				
Male	0.880	5.756	1.025–32.310	0.047
Age	0.025	1.034	0.984–1.087	0.183
Drinking	1.451	0.077	0.004–1.330	0.078
Smoking	1.092	0.255	0.408–29.471	0.255
Diabetes	1.102	0.476	0.055–4.134	0.501
Fatty liver	1.218	0.657	0.060–7.151	0.731
Coronary heart disease	1.289	0.302	0.024–3.780	
WBCs	0.089	0.924	0.775–1.100	
SF	0.002	1.009	1.004–1.014	
*Patients with OF (N* = *26)*				
Male	0.658	2.111	0.581–7.672	0.256
Age	0.020	1.042	1.001–1.084	0.044
Drinking	1.096	0.106	0.012–0.910	0.041
Smoking	0.848	0.853	0.162–4.499	0.851
Diabetes	0.973	0.322	0.048–2.168	0.244
Fatty liver	1.271	0.196	0.016–2.365	0.200
Coronary heart disease	1.265	0.152	0.013–1.812	0.136
WBCs	0.071	1.072	0.933–1.232	0.326
SF	0.002	1.011	1.006–1.015	< 0.001

*P* < 0.05 was considered statistically significant

### Prediction of poor clinical outcomes

**Fig. 3:**
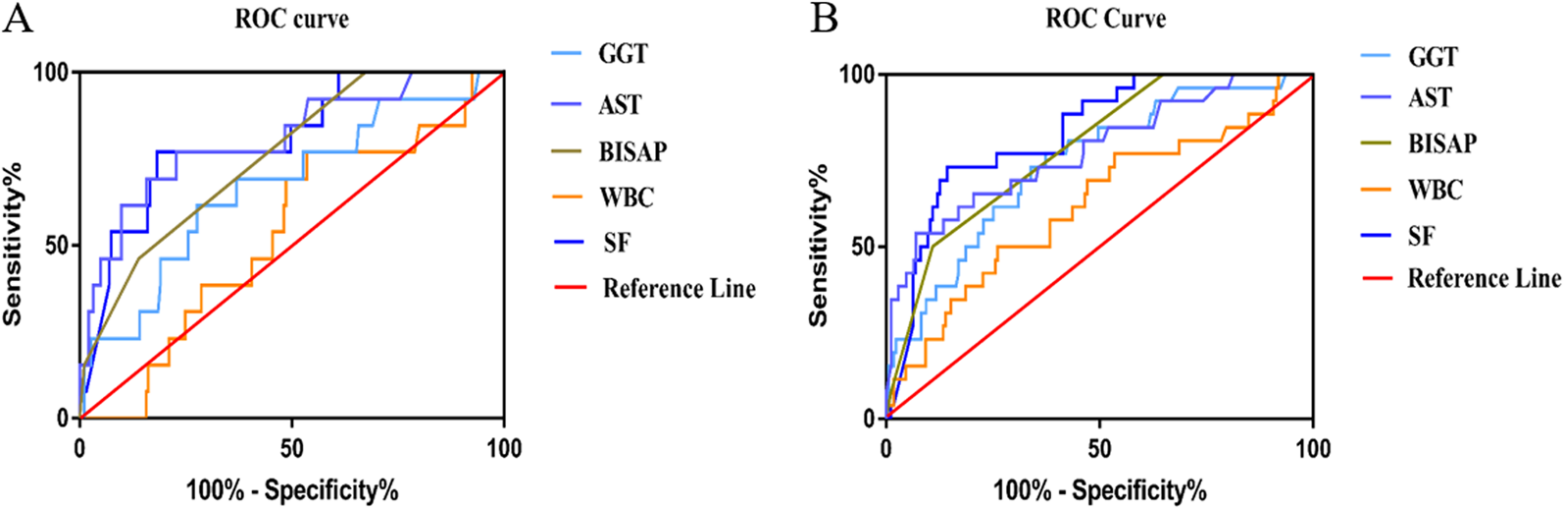
Comparison of receiver operating characteristic (ROC) curves for prediction of disease severity and organ failure since admission

As shown in Fig. 1H, the mean SF concentration was 482.3 ng/ml in patients with organ failure and 254.9 ng/ml in patients without OF; the difference was statistically significant (*P* < 0.001). The ROC curve constructed for SF is shown in Fig. 3B. The AUC values for other possible predictors of organ failure (BISAP scores; WBC; AST; GGT; Cr) are summarized in Table 3. The AUC values for the BISAP score and AST in predicting organ failure were moderate (0.758–0.729), but their sensitivity and specificity were poor. SF was superior to other indicators and had a strong predictive value (AUC 0.812, 95% CI 0.721–0.904, *P* < 0.001; best cut-off of 446.2 ng/ml; sensitivity of 0.731; specificity of 0.856).

**Table 3:** Performance of WBCs, AST, GGT,Cr, BISAP scores and SF in predicting disease severity and organ failure of AP patients

Variable	AUC	95% CI	*P* value
*SAP patients (N* = *13)*			
WBCs	0.513	0.569–0.828	0.878
AST	0.789	0.625–0.891	0.001
GGT	0.691	0.515–0.800	0.027
Cr	0.638	0.447–0.736	0.110
BISAP	0.741	0.601–0.857	0.005
SF	0.798	0.721–0.904	< 0.001
*Patients with OF (N* = *26)*			
WBCs	0.699	0.569–0.828	0.007
AST	0.758	0.625–0.891	0.001
GGT	0.658	0.515–0.800	0.033
Cr	0.592	0.447–0.736	0.215
BISAP	0.729	0.601–0.857	0.002
SF	0.812	0.721–0.904	< 0.001

*P* < 0.05 was considered statistically significant

In addition, serum SF level showed significantly greater ability for discriminating SAP from MSAP and MAP. Area under ROC curves of serum SF were remarkably larger than BISAP scores, WBC, AST, GGT and Cr, and showed higher discriminative ability over other possible predictors in predicting the occurrence of SAP (See Fig. 3A and Table 3).

## Discussion

AP is an acute inflammatory disease that is not limited to inflammation of the pancreas; rather, it involves a systemic inflammatory response. AP can induce systemic inflammatory response syndrome and multiple organ failure, such as acute lung and kidney injury, which are important causes of death in patients with SAP [20, 21]. Prevention and early identification of the occurrence of SAP are important considerations in the diagnosis and treatment of AP.

At present, Ranson’s, APECHII, BISAP, CTSI and other scores are often used to predict the severity of AP [22, 23], however, they all have disadvantages. For example, APECHII has the best forecasting performance, but the assessment is cumbersome. Ranson’s score requires 48 h to acquire the full score. The significance of the five criteria for the assessment of consciousness is not significant. The CTSI score can only be used for the assessment of local pancreatic complications. These shortcomings reduce the practicality of the above scoring methods. In recent years, with the development of laboratory technology, several serological indicators have been used to predict the severity of AP, namely, IL-6 and CRP [24, 25], however, the specific titer for the two indicators is still controversial. Therefore, it is still necessary to actively seek new, simple and economical laboratory indicators to predict the severity of AP. It is imperative to find a new biomarker that accurately predicts the severity of a patient's condition early in the onset of AP. It is important for this biomarker to both predict the patient's organ failure and also reflect the patient's systemic inflammatory response.

Iron is an essential nutrient for the body and has potential toxic effects. SF is a tissue protein that stores iron [26]. Ferritin is widely recognized as a non-specific acute-phase response and inflammation marker, reflecting the degree of oxidative stress and inflammation in vivo. Previous studies have shown that increased SF levels are usually found in the following diseases: hematological diseases (aplastic anemia, hemolytic anemia, hemochromatosis and repeated blood transfusion, etc.), malignant tumors, liver disease and acute infection [27, 28]. However, a few studies have reported the expression of SF in pancreatitis.

In this study, we examined SF levels in 200 AP patients at admission and observed the association between SF levels and the severity of acute pancreatitis.The results showed no correlation between SF and the etiology of AP. However, patients with elevated ferritin had a higher proportion of SAP and a significantly higher incidence of SIRS than patients with normal SF levels (10.9% vs. 2.8% and 44.6% vs, 25.9%, respectively). Furthermore, we analyzed the level of ferritin across disease severity in patients with AP. The results showed that SF levels were positively correlated with the Atlanta grade, Ranson’s score, CTSI score and BISAP score. These results very clearly suggest that SF is positively correlated with the severity of AP disease.

In addition, the correlations between SF levels and the other clinical serological parameters of AP patients were analyzed. As shown in Fig. 2, SF levels were positively correlated with serum ALT, AST, LDH, GGT and Cr but not with WBC, GLU or ALP. We consider the following reasons for this clinical relevance: 1. SF is mainly present in the liver, spleen, bone marrow and other reticular systems (9). When AP occurs, the destruction of hepatocytes increases, resulting in the release of large quantities of ferritin into the bloodstream [29]. Our data analysis also shows that SF levels and liver enzymes, such as ALT, AST and GGT, were positively correlated, which is consistent with the above conclusion; 2. LDH and Cr are the evaluation criteria for the Ranson’s score and are often used to assess the severity of AP. It is reasonable that SF is positively correlated with the above three indicators. However, no correlation between WBCs and SF was observed in our study. The reason for this result is not yet clear and needs further study. Besides, logistic regression analysis indicated that serum SF was independent risk factors for severe acute pancreatitis and organ failure in patients with AP. Finally, we used the ROC curve to examine the predictive value of SF for organ failure in AP. Unsurprisingly, the SF value was defined at 446.2 ng/ml with a sensitivity of 0.731 and a specificity of 0.856, and we observed the clinical characteristics in groups according to the best cut-off value of SF level (Additional file 1: Table S2), suggesting that serum ferritin may be used as an ideal biomarker to predict the severity of AP. However, the actual discriminatory abilities of SF level in a prospective patients’ cohort are still needed to be validated. Besides, studies have found that some serum biomarkers, such as circulating adipokines, serum HBDH and so on, could predict the severity and outcomes of AP [30, 31]. Nevertheless, the measurements of these biomarkers’ levels were complicated and time-consuming. The SF level of our study can be easily obtained from serum biomedical tests, which makes it easier to conduct dynamic monitoring in the daily clinical environment.

The reasons for the elevated levels of ferritin in the inflammatory response to AP are not clear, and the mechanism may be due to the process of acute pancreatitis. On the one hand, trypsin digestion produces a large number of inflammatory mediators (such as interleukin-1-beta (IL-1β), interleukin-6 (IL-6) and so on) that induce changes in the body’s iron metabolism. Under the different action of pro-inflammatory and anti-inflammatory factors, transcription or translation of ferritin can be induced. Inflammatory mediators can directly promote liver cells to release ferritin and stimulate hepcidin synthesis [32–34]. Previous studies have found that when AP occurs, hepcidin levels increase [35], thereby supporting this hypothesis. On the other hand, ferroptosis is a newly programmed cell death in which the accumulation of intracellular iron promotes lipid oxidation to cause cell death [36]. Previous studies have shown that ferroptosis is a potential therapeutic target for SAP-induced intestinal barrier injury and acute kidney injury [37, 38]. More research is needed to elucidate the role of iron metabolism in AP.

This study has several limitations. First, it is a single-center, retrospective study, and second, fewer patients were tested for ferritin. Future prospective, multi-center studies are needed to validate the obtained findings and to further explore the clinical significance of SF in AP.

## Conclusion

In brief, through this retrospective analysis, our data suggest for the first time that SF concentrations correlate positively with the severity of AP, and the quantitative assessment of SF predicts disease severity and organ failure in patients with AP.

## Supplementary Information

**Additional file 1: Table S1.** Logistic regression analysis of specific organ failures in patients with AP and serum SF levels. **Table S2.** Comparison of Clinical characteristics and outcomes between AP patients according to the cut-off value of SF level.

## Data Availability

The data used to support the findings of this study are available from the corresponding author upon request.

## Abbreviations

AP: Acute pancreatitis
SF: Serum ferritin
MAP: Mild acute pancreatitis
MSAP: Moderate severe acute pancreatitis
SAP: Severe acute pancreatitis
SIRS: Systemic inflammatory response
ARDS: Acute respiratory distress syndrome
AKI: Acute kidney injury
CT: Computed tomography
CTSI: Computed tomography severity index
BISAP: Bedside index for severity in acute pancreatitis
ALT: Alanine aminotransferase
AST: Aspartate transaminase
GGT: Glutamyl transpeptidase
ALP: Alkaline phosphatase
Cr: Creatinine
WBC: White blood cell
LDH: Lactate dehydrogenase
GLU: Glucose
ROC: Receiver operating characteristic
CI: Confidence interval
APFC: Acute peripancreatic fluid collection
ANC: Acute necrotizing collection
WON: Walled-off necrosis
SOFA: Sequential organ failure assessment
AUC: Area under the curve
