# Supplementary material for: Elevated serum ferritin levels are associated with severity and prognosis of severe acute pancreatitis: a preliminary cohort study
Source: BMC Gastroenterol. 2022 Sep 5;22:408. doi: 10.1186/s12876-022-02446-z (PMC9442953; doi:10.1186/s12876-022-02446-z)
Supplement: Supplementary file 1 — Additional file 1: Table S1. Logistic regression analysis of specific organ failures in patients with AP and serum SF levels. Table S2. Comparison of Clinical characteristics and outcomes between AP patients according to the cut-off value of SF level. [file 12876_2022_2446_MOESM1_ESM.doc]

**S Table 1. Logistic regression analysis of specific organ failures in patients with AP and serum SF levels.**

| Variable | B | OR | 95%CI | P value |
| --- | --- | --- | --- | --- |
| **Patients with OF**  ARDS  AKI  Shock | 0.010  0.008  0.005 | 1.010  1.008  1.005 | 1.005-1.016  1.002-1.014  0.999-1.010 | <0.001  0.013  0.093 |

Adjusted for variables including age, sex, drinking, smoking, diabetes, fatty liver, coronary, heart disease, WBCs. P < 0.05 was considered statistically significant.

**S Table 2.** Comparison of Clinical characteristics and outcomes between AP patients according to the cut-off value of SF level.

| Variable | Overall | SF(<446.2 ng/ml) | SF(≥446.2 ng/ml) |  |
| --- | --- | --- | --- | --- |
|  | N=200 | N=156 | N=44 | P value |
| Age (mean±sd), yrs | 57.1±15.7 | 55.9±15.1 | 61.1±17.2 | 0.053 |
| Male sex, N (%) | 110(55%) | 78(50%) | 32(72.7%) | <0.006 |
| Smoking, N (%) | 43(21.5%) | 29(18.6%) | 14(25%) | 0.087 |
| Drinking, N (%) | 29(14.5%) | 20(12.8%) | 9(17.4%） | 0.002 |
| Weight (mean±sd)  **Underlying disease, N (%)** | 70.6±12.3 | 70.5±13.0 | 71.0±10.1 | 0.856 |
| Diabetes | 19(9.5%) | 13 (8.3%) | 6 (12.0%) | 0.355 |
| Hypertension | 30(15%) | 23 (14.7%) | 7(17.4%) | 0.849 |
| Coronary heart disease | 13(6.5%) | 9 (5.8%) | 4(7.6%) | 0.432 |
| Fatty liver  **Etiology, N (%)** | 26(13%) | 21(13.5%) | 5(12.0%) | 0.716  0.337 |
| Biliary | 101(50.5%) | 76(48.7%) | 25(54.3%) |  |
| Alcohol | 14(7%) | 10(6.4%) | 4(6.5%) |  |
| Hypertriglyceridemia | 41(20.5%) | 34(21.8%) | 7(23.9%) |  |
| Others  **Severity, N (%)** | 44(22%) | 36(23.1%) | 8(15.2%) | <0.001 |
| MAP | 149(74.5%) | 128(85.9%) | 21(14.1%) |  |
| MSAP | 38(19%) | 25(65.8%) | 13(34.2%) |  |
| SAP | 13(6.5%) | 3(23.1%) | 10(76.9%) |  |
| SIRS, N (%) | 69(34.5%) | 39(25%) | 30(68.2%) | <0.001 |
| Ranson score | 1.5±1.0 | 1.2±0.9 | 2.4±0.9 | <0.001 |
| CTSI score | 2.1±1.2 | 1.8±1.0 | 3.0±1.5 | <0.001 |
| BISAP score | 0.9±0.7 | 0.7±0.7 | 1.4±0.7 | <0.001 |
| Local complications, N (%) | 28(14%) | 19(12.2%) | 9(20.5%) | 0.221 |
| Systemic complications, N (%) | 10 (5%) | 5(3.2%) | 5(11.4%) | 0.112 |

Data are presented as the means ± standard deviation or N%.P values were determined by Student’s t-test for continuous variables and the chi-square test for categorical variables.
